# The In Vitro Effects of Choline on Non-Esterified Fatty Acid-Treated Bovine Peripheral Blood Leukocytes

**DOI:** 10.3390/ani15121814

**Published:** 2025-06-19

**Authors:** Cheng-Yan Li, Yueh-Tung Chen, Tossapol Moonmanee, Jacky Peng-Wen Chan, Chien-Kai Wang

**Affiliations:** 1 Department of Animal Science, National Chung Hsing University, Taichung 402202, Taiwan; beagleis0912@hotmail.com (C.-Y.L.); flowerbud0311@gmail.com (Y.-T.C.); 2 The iEGG and Animal Biotechnology Center, National Chung Hsing University, Taichung 402202, Taiwan; 3 Northern Region Branch, Taiwan Livestock Research Institute, Ministry of Agriculture, Miaoli 368003, Taiwan; 4 Department of Animal and Aquatic Sciences, Faculty of Agriculture, Chiang Mai University, Chiang Mai 50200, Thailand; 5 Functional Feed Innovation Center, Faculty of Agriculture, Chiang Mai University, Chiang Mai 50200, Thailand; 6 Department of Veterinary Medicine, National Chung Hsing University, Taichung 402202, Taiwan

**Keywords:** non-esterified fatty acids (NEFA), choline, oxidative stress, peripheral blood leukocytes, pro-inflammatory, transition period

## Abstract

The transition period is defined as 3 weeks around parturition, involving the rapid increase in the energy demand, promoting adipose mobilization and non-esterified fatty acid (NEFA) release. High NEFA levels might cause oxidative stress and associated health risks, including the disruption of the immune capability of peripheral leukocytes. Nutrient supplementation of choline, which improves cellular lipid metabolism and controls lipid oxidation, potentially maintains the integrity of peripheral leukocytes and alleviates the impacts of increased NEFAs. This study investigated the effects of choline on bovine peripheral blood leukocytes (PBLs) treated with high levels of NEFAs. Peripheral blood mononuclear cells (PBMCs) and polymorphonuclear leukocytes (PMNs) were isolated from dry cows, and treated with 1 mM NEFA in combination with 0, 4, or 12 μM choline. The expression of pro-inflammatory cytokines and oxidative stress indicators was determined. This study demonstrates that 1 mM NEFA induces lipid oxidation and pro-inflammatory cytokine expression in PBLs. Supplementation with 4 μM and 12 μM choline significantly reduced NEFA-induced lipid oxidation; however, it did not affect pro-inflammatory cytokine mRNA expression. In conclusion, choline supplementation may help alleviate NEFA-induced oxidative stress in bovine peripheral blood leukocytes, highlighting its potential as a functional supplement during the transition period. However, it had no observable effect on mitigating pro-inflammatory responses, indicating that additional strategies may be needed to address NEFA-induced immune activation.

## Simple Summary

To simulate the immune status of dairy cows during the transition period, 1 mM non-esterified fatty acids (NEFAs) were used to induce inflammatory responses and oxidative stress in bovine immune cells. Choline supplementation was used to evaluate its potential modulatory effects. NEFA stimulation appeared to elevate the expression of pro-inflammatory cytokines at mRNA levels, along with the increased production of oxidative stress markers such as malondialdehyde (MDA). Choline supplementation significantly reduced MDA in both peripheral blood mononuclear cells (PBMCs) and polymorphonuclear leukocytes (PMNs). These observations suggest that choline might exert an alleviating effect on NEFA-induced oxidative stress, but not on pro-inflammatory cytokine expression in bovine leukocytes. Therefore, this study suggests that choline may serve as a functional supplement to alleviate oxidative stress in transition cows, supporting herd health and potentially improving overall productivity.

## 1. Introduction

The transition period of dairy cows is generally defined as three weeks before to three weeks after parturition. In this period, cows move from pregnancy and the dry period to substantial milk production. Therefore, cows experience significant physiological changes and health risks during this period [1]. During this period, dairy cows often experience a reduction in feed intake. However, to meet the increasing energy demands associated with rapid fetal development and lactation, cows might mobilize body fat reserves, which results in a state of negative energy balance (NEB). NEB results in elevated levels of serum non-esterified fatty acids (NEFAs) to satisfy the energy demands. NEFAs are free fatty acids that are not bound with glycerol. When dietary energy intake is insufficient to meet physiological demands, NEFAs are released from adipose tissue into the bloodstream through lipolysis. As the major component of triglycerides, NEFAs are mobilized during NEB to provide alternative energy substrates, primarily for hepatic β-oxidation and ketogenesis [2]. Therefore, circulating NEFA concentrations reflect the extent of fat mobilization and serve as a reliable biomarker of NEB status in dairy cows [3]. During the lactation and dry periods, the serum levels of NEFAs are less than 0.2 mM, while during the transition period, NEFA is gradually increased to 0.5 to 1 mM [4]. After parturition, NEFA reaches a peak of approximately 0.8 to 1.2 mM [3]. If NEFA levels in the prepartum period is higher than 0.5–1 mM, it is associated with an increased risk of postpartum placental retention and metritis [5]. Elevated NEFA concentrations serve as indicators of excessive lipid mobilization and negative energy balance, both of which are associated with metabolic disorders and immune disruptions in dairy cattle [6,7]. These disruptions impair the function of peripheral blood leukocytes (PBLs), reducing their amounts and capacity to proliferate, migrate, and phagocytose pathogens effectively [8,9,10], and it has been confirmed that NEFAs can induce oxidative stress and inflammation in bovine PBLs [11]. Therefore, the inflammatory responses, combined with oxidative stress, compromise immune regulation, making cows more susceptible to health risks of mastitis, metritis, and metabolic disorders, including fatty liver and ketosis [12].

Elevated serum NEFA levels under severe negative energy balance (NEB) may activate inflammatory signaling pathways through Toll-like receptor 4 (TLR4) and nuclear factor kappa B (NF-κB), leading to the increased production of pro-inflammatory cytokines such as interleukin-1β (IL-1β), IL-6, and tumor necrosis factor-α (TNF-α) [13,14,15,16,17]. This excessive inflammatory response disrupts immune homeostasis during the transition period and contributes to oxidative stress, leukocyte dysfunction, and tissue damage [18,19]. Consequently, elevated NEFA concentrations impair immune regulation and increase disease susceptibility in periparturient dairy cows [20,21,22,23].

Choline plays a crucial role in alleviating oxidative stress and inflammatory responses [24]. As a precursor for phosphatidylcholine, a vital component of cell membranes, choline supports cellular membrane integrity and prevents lipid peroxidation [25,26]. Although choline is present in the diet and can be synthesized endogenously, increased demand during the transition period and insufficient intake of precursors may limit endogenous production, making choline potentially limiting during high-demand periods such as the transition period. Therefore, choline supplements in the diet of dairy cows could be a crucial method to promote lipid metabolism [27,28]. While choline has been shown to influence lipid metabolism in hepatic cells [29], its role in immune regulation remains less defined. Previous evidence suggested that NEFA might accumulate during NEB, activating immune responses through pattern recognition receptors such as TLR4, initiating downstream inflammatory pathways [14,15]. Together, the supplement of choline may have beneficial effects on cows during the transition period. However, the exact mechanisms by which NEFAs regulate immune function in bovine leukocytes remain unclear. On the other hand, it is not clear whether oxidative stress is involved in the immune modulation. Therefore, in this study, choline was used as a potential modulator to evaluate whether it could mitigate NEFA-induced immune responses and oxidative stress in bovine peripheral blood leukocytes in vitro.

The primary objective of this study is to investigate the effects of choline on bovine PBMCs and PMNs exposed to NEFAs, a condition associated with severe NEB during the transition period. Oxidative stress and pro-inflammatory cytokine expression induced by NEFAs in choline-treated bovine PBMCs and PMNs were evaluated to elucidate the protective role of choline against NEFAs.

## 2. Materials and Methods

### 2.1. Animals and PBMC and PMN Isolation

This study was conducted from 2021 to 2022 and was approved by the IACUC of the National University (Certificate No. IACUC-109-150). Blood samples were collected from healthy Holstein cows (parities 2–3), with an average body weight of approximately 650 ± 25 kg and a body condition score (BCS) ranging from 3.0 to 3.5, at the National Chung Hsing University farm. Cows were housed in free-stall facilities, milked twice daily, and fed a diet of 50% Bermuda hay, 50% Pangola hay, and 1.5 kg of concentrate feed (20.4% crude protein and 20.3% neutral detergent fiber) twice daily. In order to minimize the metabolic disruption during the prepartum period (4 weeks before the expected calving day) and the drying-off period (8 weeks before the expected calving day), the blood samples were collected from cows during the 4–6 weeks before the calving day. The serum NEFA levels remained stable during the 4–6 week period before calving [30]. Blood (100 mL) was drawn 4–6 weeks prepartum into EDTA-coated tubes and processed within 2 hr. Samples were maintained at 4 °C during transport. Blood samples were diluted 1:1 with 1X Hank’s Balanced Salt Solution (HBSS) (No. 14175095, Thermo Fisher Scientific, Waltham, MA, USA) at room temperature. PBMCs and PMNs were isolated by density gradient centrifugation using Lymphoprep ^TM^ 1.077 (No. 1114547, Axis-Shield, Oslo, Norway). After 400× *g* 30 min centrifugation, the PBMCs were collected from the buffy coat layer located at the ficoll interface. Erythrocytes were removed by lysis buffer, according to Horn et al. (2008) [31], with a fivefold volume of erythrocyte lysis buffer, comprising 8.26 g ammonium chloride (12125-02-9, Honeywell International Inc., Charlotte, CA, USA), 1 g potassium bicarbonate (40195, Alfa Aesar, Haverhill, MA, USA), and 0.037 g Ethylenediaminetetraacetic acid (E6635, Sigma-Aldrich, Saint Louis, MO, USA) dissolved in 1 L deionized distilled water), and cells were washed with HBSS. The viability of the purified PBMCs and PMNs were detected to ensure ≥95%, and to confirm that the cell sizes of the PBMCs were within 7–9 µm and PMNs were within 10–12 µm. The cells were resuspended in CMRL medium, w/o choline chloride medium (C5900-07, USBiological, Salem, MA, USA), for subsequent experiments.

### 2.2. Immune Cell Culture and NEFA–Choline Treatment

**Figure 1:**
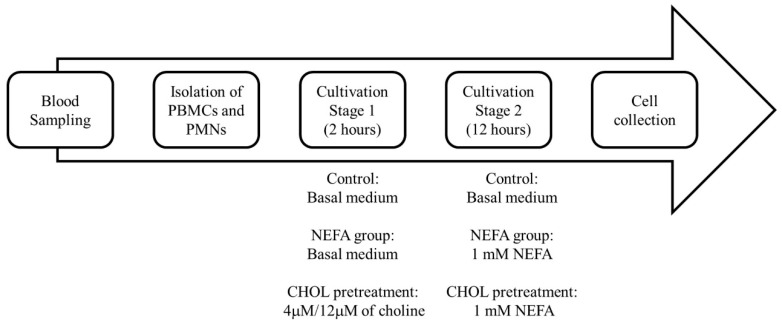
Culture process diagram.

Isolated PBMC or PMN cells were cultured as the control group in choline-free CMRL-1066 medium supplemented with 1% penicillin–streptomycin (Biological Industries, Beit HaEmek, Israel), 2% bovine serum albumin (A8806, Sigma-Aldrich, St. Louis, MO, USA), and 0.2% sodium bicarbonate (SO01310500, Scharlab S. L., Barcelona, Spain), without the addition of either NEFA or choline. To prevent disruption from the choline in the medium, the choline-free CMRL-1066 medium was chosen to culture the PBMC and PMN cells [32]. A NEFA stock solution containing 34.35 mM oleic acid (O1383, Sigma-Aldrich, Saint Louis, MO, USA), 25.2 mM palmitic acid (P0500, Sigma-Aldrich, Saint Louis, USA), and 11.4 mM stearic acid (S4751, Sigma-Aldrich, Saint Louis, MO, USA) was prepared and filtered with a 0.45 μm PVDF filter (SLHVR33RS, Millipore, Billerica, MA, USA) for sterility [33]. To clearly investigate the effects of choline on bovine immune cells, a choline-free culture medium was used in this study. In addition, since the physiological concentration of choline in dairy cows is approximately 4 µM [32], 4 µM was used to simulate normal physiological conditions, while 12 µM choline was used to mimic the supplementation of dietary choline. Choline chloride (C7527, ≥98.5% purity; Sigma-Aldrich, Darmstadt, Germany) was used to prepare the working solutions. The stock solution was freshly prepared by dissolving choline chloride in sterile distilled water and filtered through a 0.22 µm syringe filter for sterility. The stock solutions were stored at 4 °C and diluted in choline-free medium to achieve final concentrations of 4 µM and 12 µM for the treatments. PBMCs and PMNs were cultured at 4 × 10^7^ cells/well in 6-well plates (351146, Falcon, Corning Inc., New York, NY, USA) and treated with NEFA and/or choline. As mentioned above, during the transition period, the NEFA concentration may increase to 1 mM [34]. Therefore, according to Garica et al. (2018) [32], we pretreated choline for 2 h. We supplied 1 mM NEFA for 12 h to simulate in vivo conditions during this period, based on Li et al. (2024) [11]. After incubation, cells and supernatants were collected for analysis (Figure 1). Each analysis performed in this study is in triplicate.

### 2.3. Oxidative Stress Measurement

Protein concentrations were determined by a BSA standard curve (0–0.5 mg/mL) prepared via the serial dilution of a 10 mg/mL stock solution. Samples or standards (10 μL) were mixed with 190 μL of 1× Bio-Rad protein assay reagent (500-0006, Bio-Rad, Hercules, CA, USA) in a 96-well plate. After shaking for 5 min to eliminate the bubbles, absorbance was measured at OD 595 nm. A linear regression curve was generated (R^2^ > 0.99), and sample protein concentrations were calculated accordingly for SOD activity normalization.

SOD was measured by using the Superoxide Dismutase Assay Kit (706002, Cayman Chemical, Ann Arbor, MI, USA). This assay is based on the generation of superoxide radicals through the reaction of xanthine oxidase and hypoxanthine, which were subsequently detected by the reduction of tetrazolium salt to formazan dye. Then, we added a diluted radical detector and xanthine oxidase to the prepared cell lysates, and incubated the samples on a shaker and protected from light for 30 min. After incubation, the absorbance was measured at optical density (OD) 450 nm using a microplate reader, and then plotted against a standard curve to calculate the SOD activity.

Malondialdehyde (MDA), a product of lipid peroxidation, was used as a marker of oxidative stress and quantified using the Thiobarbituric Acid Reactive Substances (TBARS) Assay Kit (10009055, Cayman Chemical, Ann Arbor, MI, USA). The color reagent and the samples were added to vials and incubated in a water bath at 100 °C for 1 h. After incubation, the vials were cooled on ice for 10 min and centrifuged at 1600× *g* for 10 min; then, the supernatant was collected, and was left to equilibrate at room temperature for 30 min. A 150 µL aliquot of the supernatant was transferred to a 96-well plate, and the absorbance was measured immediately at OD 530 nm, and then plotted against a standard curve to calculate the MDA concentration.

### 2.4. Cytokine Expression by Real-Time PCR

Total RNA was extracted from the PBMCs and PMNs using TRIzol Reagent (15596023, Invitrogen, Carlsbad, CA, USA). The RNA purity and concentration were assessed using a spectrophotometer, ensuring a 260/280 nm ratio of 1.8–2.0. Reverse transcription was performed using the SuperScript IV Reverse Transcriptase kit (Invitrogen, Carlsbad, CA, USA). The synthesized cDNA was stored at −80 °C. The real-time quantitative polymerase chain reaction (qPCR) mixture included iTaqTM Universal SYBR ^®^ Green Supermix (Bio-Rad, Bio-Rad Laboratories, Hercules, CA, USA) so to confirm the gene expression in treatments. Gene expression levels were analyzed using the ΔΔCT method, with glyceraldehyde-3-phosphate dehydrogenase (GAPDH) as the reference gene (Table 1). The results provided insights into the changes in pro-inflammatory-related gene expression under NEFA stimulation and choline incubation.

### 2.5. Statistics

IBM SPSS Statistics 29 (IBM, New York, NY, USA) was used for statistical analysis. Data are presented as mean ± standard error (SE). The MDA concentration, SOD activity, and mRNA expression in the control group, NEFA group, and choline-pretreated groups were analyzed using a one-way ANOVA. Differences between treatment groups were evaluated post hoc using the LSD method. Statistical significance was set at *p* < 0.05, indicating significant differences.

## 3. Results

### 3.1. NEFA-Induced Oxidative Stress and Antioxidative Stress Effects of Choline

To determine whether 1 mM NEFA induces oxidative stress, the MDA levels and SOD activity were measured in the PBMCs and PMNs following 12 h of exposure. The results showed that 1 mM NEFA significantly increased the MDA levels in the PBMCs by 18% compared to the control group (2.85 ± 0.15 µM vs. 2.41 ± 0.15 µM; *p* < 0.01). Similarly, in the PMNs, 1 mM NEFA significantly increased the MDA levels (2.34 ± 0.26 µM vs. 1.69 ± 0.18 µM; *p* < 0.05). However, NEFA did not significantly affect the SOD activity in either the PBMCs or PMNs (*p* > 0.05) (Table 2).

Pre-incubation with 4 µM or 12 µM choline significantly reduced the NEFA-induced MDA levels in the PBMCs by 20 and 32% (*p* < 0.05), and in the PMNs by 5 and 27%, respectively (*p* < 0.05). Notably, choline restored both the PBMC and PMN MDA levels to control levels (*p* > 0.05). Choline pretreatment, however, did not affect the SOD activity in either cell type under NEFA stimulation (*p* > 0.05). These findings demonstrated that NEFA induced oxidative stress by increasing the MDA levels, which was mitigated by choline supplementation, though the SOD activity remained unaffected.

### 3.2. NEFA-Induced Inflammatory Cytokine Expression and Effects of Choline

PBMCs and PMNs isolated from cows were treated with 1 mM NEFA for 12 h, and pro-inflammatory cytokine expression was determined using qPCR. The results showed that NEFA significantly increased the mRNA expression of *IL-6* (2.76 ± 0.4) and *IL-1β* (1.78 ± 0.2) in the PBMCs (*p* < 0.05), while *IL-8*, *IL-10*, *CSF-1*, *CSF-2*, and *CSF- 3* showed no significant changes (*p* > 0.05). In the PMNs, NEFA significantly induced the mRNA expression of *IL-1β* (3.27 ± 0.88) and *CSF-2* (4.42 ± 1.38) (*p* < 0.05), but had no significant effect on *IL-6*, *IL-8*, *IL-10*, *CSF-1*, or *CSF-3* (*p* > 0.05) (Table 3).

To assess the effects of choline, cells were pre-incubated with 4 μM or 12 μM choline for 2 h before NEFA stimulation for 12 h. The results showed that neither 4 μM nor 12 μM choline pretreatment had an effect on inflammatory cytokine mRNA expression in the PBMCs and PMNs. In summary, 1 mM NEFA significantly increased the mRNA expression of inflammation-related cytokine in PBLs. Choline pretreatment did not attenuate NEFA-induced pro-inflammatory cytokine expression in either the PBMCs or PMNs (Table 3).

## 4. Discussion

NEFA generates ROS through mitochondrial β-oxidation, which might peroxidize lipids, forming MDA, an oxidative stress marker. Increased MDA was observed in the PBMCs, but there were comparatively minimal changes in the PMNs. Research has shown that there were similar PBMC responses [35,36], while PMNs showed limited lipid oxidation [37,38], possibly due to their functional role in pathogen clearance by producing ROS to eliminate pathogens [39]. Overall, these results indicate that PBMCs are more sensitive to NEFA-induced lipid peroxidation than PMNs, which may be due to distinct susceptibilities in their oxidative stress responses. Additionally, 1 mM NEFA did not affect the SOD activity in either cell types after 12 h, aligning with the findings that antioxidant responses may vary depending on stimuli [40,41,42]. Moreover, previous studies showed that bovine hepatocytes stimulated with 1.2 mM NEFA exhibited the lowest SOD activity after 1 h of stimulation, which recovered to the control group after 12 h [42]. Therefore, the stimulation duration may also be an important factor influencing SOD activity. These findings suggest that NEFA induces oxidative stress through lipid oxidation.

Choline’s role in oxidative stress was also investigated. As a precursor for acetylcholine and phosphatidylcholine, choline helps maintain membrane integrity and lipid metabolism [43], and it may help to ease the oxidative stress on cells. Choline has been reported to lower MDA levels and oxidative stress markers [44,45]. In this study, pre-incubation with choline reduced the MDA in the PBMCs and PMNs, indicating that it mitigates NEFA-induced oxidative stress. In cattle mammary epithelial cells, 100 μM choline reduced ROS and MDA under heat stress [46]. However, choline did not affect the SOD activity in NEFA-induced PBMCs or PMNs in this study. This is consistent with studies showing that choline did not affect the SOD or GSH-Px activity in vitro [46,47], but it did enhance their activity under in vivo heat stress conditions [46]. Therefore, choline’s antioxidant enzyme modulation may depend on the type of stress. Overall, choline may serve as a functional nutrient to attenuate NEFA-induced oxidative stress during the transition period.

This study demonstrated the pro-inflammatory responses induced by NEFA treatment in PBMCs and PMNs. NEFA significantly increased *IL-1β* and *IL-6* mRNA expression in the PBMCs, while upregulating *IL-1β* and *CSF-2* in the PMNs. IL-1β and IL-6 are key cytokines involved in acute inflammation. *CSF-2* upregulation in PMNs might enhance the expression of pro-inflammatory cytokines such as *IL-6* [48], influencing immune cell differentiation and altering immune homeostasis [49]. IL-1β is a pro-inflammatory cytokine that is secreted by various cells, including immune and endothelial cells, enhances immune cell proliferation, and induces the expression of other cytokines [50,51]. IL-6 functions in both pro- and anti-inflammatory responses, stimulating acute-phase protein production [52]. CSF binds to CSF receptors and affects the proliferation and differentiation of neutrophils [53]. CSF-1 acts on monocytes and macrophages, CSF-3 can regulate the proliferation and differentiation of neutrophils [54,55], and CSF-2 acts on macrophages or neutrophils to promote inflammatory responses [56]. Our findings support previous reports that NEFA induces pro-inflammatory cytokine production in a dose-dependent manner [57].

NEFA may activate TLRs and G-protein coupled receptor 40 (GPR40) receptors, triggering NF-κB signaling. Kumolosasi et al. (2014) [58] reported IL-1β upregulation in PBMCs after 4 h of inflammation induction, thereby influencing the CSF family expression. Consistently, NEFA treatment increased IL-1β protein expression [17,59,60,61]. Free fatty acids may also engage receptors, including CD26, IL-15R, programmed death-1 (PD-1), IL-33R, TLRs, IL-6R, IL-4/13R, CD36, fatty acid-binding proteins (FABPs), and free fatty acid receptor-1, thereby activating downstream pathways, including peroxisome proliferator-activated receptors (PPARs), signal transducer and activator of transcription 3 (STAT 3), forkhead box protein P3 (FoxP3), C-MYC, phospholipase C-protein kinase C (PLC/PKC), and sterol regulatory element-binding proteins (SREBPs) [15,62,63,64,65]. These pathways regulate cytokine expression and may underlie the inflammatory effects observed. The exact cellular signaling pathways activated by NEFA to upregulate pro-inflammatory responses in bovine PBMCs and PMNs require further identification.

The role of choline was also examined. In this study, pretreatment with 4 μM or 12 μM choline did not alter the pro-inflammatory cytokine mRNA expression in the NEFA-treated PBMCs and PMNs. Vailati-Riboni et al. (2017) reported that choline supplementation in dairy cows, whether supplied prepartum or postpartum, did not significantly affect the plasma concentrations of pro-inflammatory cytokines such as IL-1β and IL-6 [66]. Similar findings were reported by Zhou et al. (2016) and Swartz et al. (2023) [67,68]. In vitro evidence from Garcia et al. (2018) showed that choline supplementation in PMNs did not significantly affect the inflammatory cytokine mRNA expression, including *NF-κB* and *TNF-α* [32], but upregulated the genes involved in choline metabolism. Compared to methionine, choline may exhibit weaker anti-inflammatory effects due to its need for metabolic conversion. In vivo, choline is converted into bioactive forms like acetylcholine and phosphatidylcholine [25,69,70,71], possibly enhancing its function. In contrast, in vitro systems may require higher choline concentrations to observe similar effects [72].

Oxidative stress was indicated by the production of MDA. MDAs are by-products of lipid peroxidation, initiated by ROS attacking polyunsaturated fatty acids [73]. While oxidative stress reflects damage from ROS, inflammation involves immune receptor activation and cytokine production through the NF-κB and MAPK pathways [15,40]. TBARS indicates oxidative damage endpoints, while inflammation reflects signaling responses. These are related but distinct biological processes [74]. However, oxidative stress and inflammation may interact with each other. For example, ROS might activate cell signaling pathways such as NF-κB, leading to the production of pro-inflammatory cytokines [75]. Therefore, oxidative stress may be associated with inflammatory responses, even though their initial triggers and regulatory mechanisms are different.

Choline supplementation showed its potential in alleviating NEFA-induced disruption in bovine PBLs. NEFA, which is elevated during the transition period, affects cytokine expression and immune regulation [33,76]. In vitro, choline reduced lipid oxidation, suggesting its use in preserving the functionality of PBLs under NEFA stress. The primary culture of bovine PBLs provides a proper in vitro model for evaluating the immune cell responses in cattle. However, further studies are needed to clarify NEFA-activated signaling pathways and the optimize choline dosage and duration. Moreover, in vivo validation is essential to confirm choline’s role in immune modulation and oxidative stress reduction during the transition period in dairy cows.

## 5. Conclusions

Choline supplementation at both 4 µM and 12 µM concentrations effectively reduced NEFA-induced lipid peroxidation. However, choline did not attenuate the upregulation of inflammatory cytokine gene expression, suggesting that its protective effects are primarily associated with oxidative stress mitigation rather than immunomodulation. These results highlight choline’s potential as a functional nutrient to alleviate NEFA-induced oxidative damage during the transition period. Although its anti-inflammatory effects were not evident in our study, the antioxidant role of choline might contribute to maintaining immune cell integrity and function in transition-period cows. These results highlight choline’s potential as a functional nutrient to alleviate NEFA-induced oxidative damage during the transition period, but additional strategies may be required to control NEFA-induced inflammatory responses during the transition period.

## Figures and Tables

**Table 1:** The qPCR primer sequences.

Gene Symbol	GeneBank Accession Number	Forward Primer 5′-3′	Product Size (bp)
		Reverse Primer 3′-5′	
*GAPDH*	NM_001034034.2	CAAGCTCATTTCCTGGTACGAC	130
		AACTCTTCCTCTCGTGCTCC	
*IL-1β*	NM_174093	GACGAGTTTCTGTGTGACGC	149
		ATGCAGAACACCACTTCTCGG	
*IL-6*	NM_173923.2	TGAAAGCAGCAAGGAGACACT	99
		CAAATCGCCTGATTGAACCCAG	
*IL-10*	NM_174088.1	CTGTTGACCCAGTCTCTGCT	216
		GCTCTTGTTTTCGCAGGGC	
*CSF-1*	NM_174026.1	GCCCGTTTTAACTCCGTTCC	180
		TGGCTCTTGATGGCTCCGAC	
*CSF-2*	NM_174027.2	GGCCACCCACTACGAGAAAC	160
		CTGGTTTGGCCTGCTTCACT	
*CSF-3*	NM_174028.1	GCCTGAACCAACTACACGGC	209
		GGCTGAAGTGAAGGTCGGCA	

Abbreviation: GAPDH, glyceraldehyde 3-phosphate dehydrogenase; IL-1β/6/10, interleukin-1β/6/10; CSF-1/2/3, colony-stimulating factors-1/2/3.

**Table 2:** Effect of NEFA and choline pretreatment combined with NEFA on the PBMC and PMN oxidative stress indicators.

Oxidative Stress Indicators	Control	1 mM NEFA	1 mM NEFA + 4 µM CHOL	1 mM NEFA + 12 µM CHOL
PBMC				
TBARS (µM/10^7^ cells)	2.41 ± 0.15 ^a^	2.85 ± 0.15 ^b^ (18%)	2.15 ± 0.19 ^a^ (32%)	2.38 ± 0.16 ^a^ (20%)
SOD (U/mL)	2.65 ± 0.34 ^a^	3.08 ± 0.34 ^a^	2.89 ± 0.24 ^a^	2.92 ± 0.41 ^a^
PMN				
TBARS (µM/10^7^ cells)	1.69 ± 0.18 ^a^	2.34 ± 0.26 ^b^ (38%)	2.22 ± 0.14 ^a^ (5%)	1.84 ± 0.24 ^a^ (27%)
SOD(U/mL)	0.86 ± 0.17 ^a^	0.91 ± 0.2 ^a^	0.8 ± 0.15 ^a^	0.87 ± 0.17 ^a^

Data are presented as mean ± standard error. TBARS sample size = 12; SOD sample size = 10. The different letters indicate significant differences among treatments (*p* < 0.05). Control: basal culture medium treatment 2 h + basal culture medium treatment 12 h; 1 mM NEFA: basal culture medium treatment 2 h + 1 mM NEFA treatment 12 h; 1 mM NEFA+ 4 μM/12μM CHOL: 4 μM/12 μM choline pretreatment 2 h + 1 mM NEFA treatment 12 h. Abbreviation: SOD, superoxide dismutase; TBARS, thiobarbituric acid reactive substances.

**Table 3:** Effect of NEFA and choline pretreatment combined with NEFA on the PBMC and PMN cytokine gene expression.

	PBMC				PMN			
Gene	Control	1 mM NEFA	1 mM NEFA+ 4 µM CHOL	1 mM NEFA+ 12 µM CHOL	Control	1 mM NEFA	1 mM NEFA + 4 µM CHOL	1 mM NEFA + 12 µM CHOL
*IL-1β*	1 ± 0.37 ^a^	1.78 ± 0.2 ^b^	1.81 ± 0.27 ^b^	2.28 ± 0.29 ^b^	1 ± 0.58 ^a^	3.27 ± 0.88 ^b^	2.87 ± 0.87 ^ab^	2.71 ± 0.51 ^ab^
*IL-6*	1 ± 0.36 ^a^	2.76 ± 0.4 ^b^	2.63 ± 0.46 ^b^	2.71 ± 0.61 ^b^	1 ± 0.5 ^a^	1.58 ± 0.3 ^a^	2.11 ± 0.39 ^a^	4.27 ± 2.78 ^a^
*IL-8*	1 ± 0.57 ^a^	2.08 ± 0.49 ^a^	2.03 ± 0.54 ^a^	2.62 ± 0.93 ^a^	1 ± 0.42 ^a^	5.09 ± 3.53 ^a^	1.73 ± 0.35 ^a^	1.5 ± 0.19 ^a^
*IL-10*	1 ± 0.3 ^a^	1.73 ± 0.43 ^ab^	2.1 ± 0.5 ^b^	1.72 ± 0.33 ^ab^	1 ± 0.43 ^a^	2.14 ± 0.51 ^ab^	2.08 ± 0.57 ^ab^	2.62 ± 0.75 ^b^
*CSF-1*	1 ± 0.46 ^a^	0.69 ± 0.15 ^a^	0.72 ± 0.13 ^a^	2.85 ± 2.24 ^a^	1 ± 0.33 ^a^	1.43 ± 0.35 ^a^	1.89 ± 0.95 ^a^	1.18 ± 0.39 ^a^
*CSF-2*	1 ± 0.53 ^a^	0.93 ± 0.18 ^a^	1.25 ± 0.21 ^a^	1.01 ± 0.28 ^a^	1 ± 0.59 ^a^	4.42 ± 1.38 ^b^	3.18 ± 1.05 ^ab^	2.71 ± 0.86 ^ab^
*CSF-3*	1 ± 0.88 ^a^	7.96 ± 2.39 ^ab^	12.33 ± 3.88 ^b^	13.34 ± 3.95 ^b^	1 ± 0.73 ^a^	5.76 ± 2.18 ^a^	5.49 ± 1.9 ^a^	5.38 ± 1.89 ^a^

Data are presented as mean ± standard error. Sample size = 7. Different letters among treatments indicate *p* < 0.05. Control: basal culture medium treatment 2 h + basal culture medium treatment 12 h; 1 mM NEFA: basal culture medium treatment 2 h +1 mM NEFA treatment 12 h; 1 mM NEFA+ 4 μM/12 μM CHOL: 4 μM/12 μM choline pretreatment 2 h +1 mM NEFA treatment 12 h. Abbreviation: IL-1β/6/10, interleukin-1β/6/10; CSF-1/2/3, colony-stimulating factors-1/2/3.

## Data Availability

The raw data supporting the conclusions of this article will be made available by the authors on request.

## References

[1] Grummer R.R. (1995). Impact of changes in organic nutrient metabolism on feeding the transition dairy cow. J. Anim. Sci..

[2] Li P., Liu Y., Zhang Y., Long M., Guo Y., Wang Z., Li X., Zhang C., Li X., He J. (2013). Effect of non-esterified fatty acids on fatty acid metabolism-related genes in calf hepatocytes cultured in vitro. Cell. Physiol. Biochem..

[3] Adewuyi A., Gruys E., Van Eerdenburg F. (2005). Non esterified fatty acids (NEFA) in dairy cattle. A review. Vet. Q..

[4] Contreras G., O’boyle N., Herdt T., Sordillo L. (2010). Lipomobilization in periparturient dairy cows influences the composition of plasma nonesterified fatty acids and leukocyte phospholipid fatty acids. J. Dairy Sci..

[5] Chapinal N., Carson M., Duffield T., Capel M., Godden S., Overton M., Santos J., LeBlanc S. (2011). The association of serum metabolites with clinical disease during the transition period. J. Dairy Sci..

[6] Ospina P., Nydam D., Stokol T., Overton T. (2010). Evaluation of nonesterified fatty acids and β-hydroxybutyrate in transition dairy cattle in the northeastern United States: Critical thresholds for prediction of clinical diseases. J. Dairy Sci..

[7] Sharma N., Singh N., Singh O., Pandey V., Verma P. (2011). Oxidative stress and antioxidant status during transition period in dairy cows. Asian-Australas. J. Anim. Sci..

[8] Herr M., Bostedt H., Failing K. (2011). IgG and IgM levels in dairy cows during the periparturient period. Theriogenology.

[9] Saed H.A., Ibrahim H.M., El-Khodery S.A., Youssef M.A. (2020). Relationship between expression pattern of vitamin D receptor, 1 alpha-hydroxylase enzyme, and chemokine RANTES genes and selected serum parameters during transition period in Holstein dairy cows. Vet. Rec. Open.

[10] Trevisi E., Amadori M., Archetti I., Lacetera N., Bertoni G. (2011). Inflammatory response and acute phase proteins in the transition period of high-yielding dairy cows. Acute Phase Proteins as Early Non-Specific Biomarkers of Human and Veterinary Diseases.

[11] Li C.-Y., Lin W.-C., Moonmanee T., Chan J.P.-W., Wang C.-K. (2024). The protective role of vitamin E against oxidative stress and immunosuppression induced by non-esterified fatty acids in bovine peripheral blood leukocytes. Animals.

[12] Sordillo L., Mavangira V. (2014). The nexus between nutrient metabolism, oxidative stress and inflammation in transition cows. Anim. Prod. Sci..

[13] Baeuerle P.A., Henkel T. (1994). Function and activation of NF-kappa B in the immune system. Annu. Rev. Immunol..

[14] Francaux M. (2009). Toll-like receptor signalling induced by endurance exercise. Appl. Physiol. Nutr. Metab..

[15] Mena J., Manosalva C., Ramirez R., Chandia L., Carroza D., Loaiza A., Burgos R.A., Hidalgo M.A. (2013). Linoleic acid increases adhesion, chemotaxis, granule release, intracellular calcium mobilisation, MAPK phosphorylation and gene expression in bovine neutrophils. Vet. Immunol. Immunopathol..

[16] Akira S., Takeda K. (2004). Toll-like receptor signalling. Nat. Rev. Immunol..

[17] Zhang Y., Li X., Zhang H., Zhao Z., Peng Z., Wang Z., Liu G., Li X. (2018). Non-esterified fatty acids over-activate the TLR2/4-NF-κb signaling pathway to increase inflammatory cytokine synthesis in neutrophils from ketotic cows. Cell. Physiol. Biochem..

[18] Bradford B., Yuan K., Farney J., Mamedova L., Carpenter A. (2015). Invited review: Inflammation during the transition to lactation: New adventures with an old flame. J. Dairy Sci..

[19] Pascottini O.B., Van Schyndel S., Spricigo J., Carvalho M., Mion B., Ribeiro E., LeBlanc S. (2020). Effect of anti-inflammatory treatment on systemic inflammation, immune function, and endometrial health in postpartum dairy cows. Sci. Rep..

[20] Crookenden M., Heiser A., Murray A., Dukkipati V., Kay J., Loor J., Meier S., Mitchell M., Moyes K., Walker C. (2016). Parturition in dairy cows temporarily alters the expression of genes in circulating neutrophils. J. Dairy Sci..

[21] Hammon D., Evjen I., Dhiman T., Goff J., Walters J. (2006). Neutrophil function and energy status in Holstein cows with uterine health disorders. Vet. Immunol. Immunopathol..

[22] Hoeben D., Monfardini E., Opsomer G., Burvenich C., Dosogne H., De Kruif A., Beckers J.-F. (2000). Chemiluminescence of bovine polymorphonuclear leucocytes during the periparturient period and relation with metabolic markers and bovine pregnancy-associated glycoprotein. J. Dairy Res..

[23] Kehrli M.E., Nonnecke B.J., Roth J.A. (1989). Alterations in bovine neutrophil function during the periparturient period. Am. J. Vet. Res.

[24] Huang S.-Y., Yang Z.-J., Cheng J., Li H.-Y., Chen S., Huang Z.-H., Chen J.-D., Xiong R.-G., Yang M.-T., Wang C. (2025). Choline alleviates cognitive impairment in sleep-deprived young mice via reducing neuroinflammation and altering phospholipidomic profile. Redox Biol..

[25] Goselink R., Van Baal J., Widjaja H., Dekker R., Zom R., De Veth M., Van Vuuren A. (2013). Effect of rumen-protected choline supplementation on liver and adipose gene expression during the transition period in dairy cattle. J. Dairy Sci..

[26] Yao N., Li W., Xu G., Duan N., Yu G., Qu J. (2023). Choline metabolism and its implications in cancer. Front. Oncol..

[27] Santos J.E.P., Lima F.S. Feeding rumen-protected choline to transition dairy cows. Proceedings of the 20th Annual Florida Ruminant Nutrition Symposium.

[28] Grummer R. Choline: A limiting nutrient for transition dairy cows. Proceedings of the Cornell Nutrition Conference.

[29] Jiang X., Greenwald E., Jack-Roberts C. (2016). Effects of choline on DNA methylation and macronutrient metabolic gene expression in in vitro models of hyperglycemia. Nutr. Metab. Insights.

[30] Rastani R., Grummer R., Bertics S., Gümen A., Wiltbank M., Mashek D., Schwab M. (2005). Reducing dry period length to simplify feeding transition cows: Milk production, energy balance, and metabolic profiles. J. Dairy Sci..

[31] Horn P., Bork S., Horn P., Bork S., Diehlmann A., Walenda T., Eckstein V., Ho A., Wagner W. (2008). Isolation of human mesenchymal stromal cells is more efficient by red blood cell lysis. Cytotherapy.

[32] Garcia M., Mamedova L.K., Barton B., Bradford B.J. (2018). Choline regulates the function of bovine immune cells and alters the mRNA abundance of enzymes and receptors involved in its metabolism in vitro. Front. Immunol..

[33] Ster C., Loiselle M.-C., Lacasse P. (2012). Effect of postcalving serum nonesterified fatty acids concentration on the functionality of bovine immune cells. J. Dairy Sci..

[34] Grummer R.R. (1993). Etiology of lipid-related metabolic disorders in periparturient dairy cows. J. Dairy Sci..

[35] Campoio T., Oliveira F., Otton R. (2011). Oxidative stress in human lymphocytes treated with fatty acid mixture: Role of carotenoid astaxanthin. Toxicology Vitr..

[36] Volpe C.M.O., Abreu L.F.M., Gomes P.S., Gonzaga R.M., Veloso C.A., Nogueira-Machado J.A. (2014). The production of nitric oxide, IL-6, and TNF-alpha in palmitate-stimulated PBMNCs is enhanced through hyperglycemia in diabetes. Oxidative Med. Cell. Longev..

[37] Rayaman P., Rayaman E., Çevikbas A., Demirtunç R., Sehirli A.O., Gürer Ü.S. (2013). The effect of some antibiotics on Polymorphonuclear Leukocyte (PMN) functions and PMN’S myeloperoxidase activity, glutathione and malondialdehyde levels of patients with type 2 diabetes mellitus in vitro. Clin. Exp. Health Sci..

[38] Reyes-Quiroz M.E., Alba G., Saenz J., Santa-María C., Geniz I., Jiménez J., Ramírez R., Martín-Nieto J., Pintado E., Sobrino F. (2014). Oleic acid modulates mRNA expression of liver X receptor (LXR) and its target genes ABCA1 and SREBP1c in human neutrophils. Eur. J. Nutr..

[39] Johansson Å.C., Ohlsson S., Pettersson Å., Bengtsson A.A., Selga D., Hansson M., Hellmark T. (2016). Impaired phagocytosis and reactive oxygen species production in phagocytes is associated with systemic vasculitis. Arthritis Res. Ther..

[40] Gao W., Du X., Lei L., Wang H., Zhang M., Wang Z., Li X., Liu G., Li X. (2018). NEFA-induced ROS impaired insulin signalling through the JNK and p38MAPK pathways in non-alcoholic steatohepatitis. J. Cell. Mol. Med..

[41] Li P., Li L., Zhang C., Cheng X., Zhang Y., Guo Y., Long M., Yang S., He J. (2019). Palmitic acid and β-hydroxybutyrate induce inflammatory responses in bovine endometrial cells by activating oxidative stress-mediated NF-κB signaling. Molecules.

[42] Zhang B., Li M., Yang W., Loor J.J., Liang Y., Wang S., Zhao Y., Guo H., Ma X., Yu L. (2020). Mitochondrial dysfunction and endoplasmic reticulum stress in calf hepatocytes are associated with fatty acid-induced ORAI calcium release-activated calcium modulator 1 signaling. J. Dairy Sci..

[43] EFSA Panel on Dietetic Products, Nutrition and Allergies (2011). Scientific Opinion on the substantiation of health claims related to choline and contribution to normal lipid metabolism (ID 3186), maintenance of normal liver function (ID 1501), contribution to normal homocysteine metabolism (ID 3090), maintenance of normal neurological function (ID 1502), contribution to normal cognitive function (ID 1502), and brain and neurological development (ID 1503) pursuant to Article 13 (1) of Regulation (EC) No 1924/2006. Eur. Food Saf. Auth. J..

[44] Adjoumani J.J.Y., Abasubong K.P., Phiri F., Xu C., Liu W., Zhang D. (2019). Effect of dietary betaine and choline association on lipid metabolism in blunt snout bream fed a high-fat diet. Aquac. Nutr..

[45] Cetinkaya M., Cansev M., Cekmez F., Tayman C., Canpolat F.E., Kafa I.M., Uysal S., Tunc T., Sarici S.U. (2013). CDP-choline reduces severity of intestinal injury in a neonatal rat model of necrotizing enterocolitis. J. Surg. Res..

[46] Yang M., Kuang M., Wang G., Ali I., Tang Y., Yang C., Li Y., Li L. (2021). Choline attenuates heat stress-induced oxidative injury and apoptosis in bovine mammary epithelial cells by modulating PERK/Nrf-2 signaling pathway. Mol. Immunol..

[47] Zhu J., Wu Y., Tang Q., Leng Y., Cai W. (2014). The effects of choline on hepatic lipid metabolism, mitochondrial function and antioxidative status in human hepatic C3A cells exposed to excessive energy substrates. Nutrients.

[48] Shiomi A., Usui T. (2015). Pivotal roles of GM-CSF in autoimmunity and inflammation. Mediat. Inflamm..

[49] Bhattacharya P., Thiruppathi M., Elshabrawy H.A., Alharshawi K., Kumar P., Prabhakar B.S. (2015). GM-CSF: An immune modulatory cytokine that can suppress autoimmunity. Cytokine.

[50] Dinarello C.A. (1996). Biologic basis for interleukin-1 in disease. Blood.

[51] Voronov E., Dotan S., Krelin Y., Song X., Elkabets M., Carmi Y., Rider P., Cohen I., Romzova M., Kaplanov I. (2013). Unique versus redundant functions of IL-1α and IL-1β in the tumor microenvironment. Front. Immunol..

[52] Gabay C. (2006). Interleukin-6 and chronic inflammation. Arthritis Res. Ther..

[53] Solaroglu I., Cahill J., Jadhav V., Zhang J.H. (2006). A novel neuroprotectant granulocyte-colony stimulating factor. Stroke.

[54] Basu S., Dunn A., Ward A. (2002). G-CSF: Function and modes of action. Int. J. Mol. Med..

[55] Chitu V., Stanley E.R. (2006). Colony-stimulating factor-1 in immunity and inflammation. Curr. Opin. Immunol..

[56] Hamilton J.A. (2019). GM-CSF in inflammation. J. Exp. Med..

[57] Li C.-Y., Liao Y.-W., Liu C.-S., Cheng C.-Y., Chan J.P.-W., Wang C.-K. (2021). In vitro effects of nonesterified fatty acids and β-hydroxybutyric acid on inflammatory cytokine expression in bovine peripheral blood leukocytes. Ital. J. Anim. Sci..

[58] Kumolosasi E., Salim E., Jantan I., Ahmad W. (2014). Kinetics of intracellular, extracellular and production of pro-inflammatory cytokines in lipopolysaccharide-stimulated human peripheral blood mononuclear cells. Trop. J. Pharm. Res..

[59] Ma X.-Z., Pang Z.-D., Wang J.-H., Song Z., Zhao L.-M., Du X.-J., Deng X.-L. (2018). The role and mechanism of KCa3. 1 channels in human monocyte migration induced by palmitic acid. Exp. Cell Res..

[60] Tian H., Liu C., Zou X., Wu W., Zhang C., Yuan D. (2015). MiRNA-194 regulates palmitic acid-induced toll-like receptor 4 inflammatory responses in THP-1 cells. Nutrients.

[61] Snodgrass R.G., Huang S., Choi I.-W., Rutledge J.C., Hwang D.H. (2013). Inflammasome-mediated secretion of IL-1β in human monocytes through TLR2 activation; modulation by dietary fatty acids. J. Immunol..

[62] Manosalva C., Mena J., Velasquez Z., Colenso C.K., Brauchi S., Burgos R.A., Hidalgo M.A. (2015). Cloning, identification and functional characterization of bovine free fatty acid receptor-1 (FFAR1/GPR40) in neutrophils. PLoS ONE.

[63] Mena S.J., Manosalva C., Carretta M.D., Teuber S., Olmo I., Burgos R.A., Hidalgo M.A. (2016). Differential free fatty acid receptor-1 (FFAR1/GPR40) signalling is associated with gene expression or gelatinase granule release in bovine neutrophils. Innate Immun..

[64] Wu X., Schauss A.G. (2012). Mitigation of inflammation with foods. J. Agric. Food Chem..

[65] Zhang S., Lv K., Liu Z., Zhao R., Li F. (2024). Fatty acid metabolism of immune cells: A new target of tumour immunotherapy. Cell Death Discov..

[66] Vailati-Riboni M., Zhou Z., Jacometo C., Minuti A., Trevisi E., Luchini D., Loor J. (2017). Supplementation with rumen-protected methionine or choline during the transition period influences whole-blood immune response in periparturient dairy cows. J. Dairy Sci..

[67] Zhou Z., Bulgari O., Vailati-Riboni M., Trevisi E., Ballou M., Cardoso F., Luchini D., Loor J. (2016). Rumen-protected methionine compared with rumen-protected choline improves immunometabolic status in dairy cows during the peripartal period. J. Dairy Sci..

[68] Swartz T.H., Bradford B., Mamedova L., Estes K. (2023). Effects of dietary rumen-protected choline supplementation to periparturient dairy cattle on inflammation, metabolism, and performance during an intramammary lipopolysaccharide challenge. J. Dairy Sci..

[69] Coleman D.N., Vailati-Riboni M., Elolimy A.A., Cardoso F.C., Rodriguez-Zas S.L., Miura M., Pan Y.-X., Loor J.J. (2019). Hepatic betaine-homocysteine methyltransferase and methionine synthase activity and intermediates of the methionine cycle are altered by choline supply during negative energy balance in Holstein cows. J. Dairy Sci..

[70] Zhou Z., Ferdous F., Montagner P., Luchini D., Correa M., Loor J. (2018). Methionine and choline supply during the peripartal period alter polymorphonuclear leukocyte immune response and immunometabolic gene expression in Holstein cows. J. Dairy Sci..

[71] Mehta A.K., Singh B.P., Arora N., Gaur S.N. (2010). Choline attenuates immune inflammation and suppresses oxidative stress in patients with asthma. Immunobiology.

[72] Lopreiato V., Vailati-Riboni M., Bellingeri A., Khan I., Farina G., Parys C., Loor J. (2019). Inflammation and oxidative stress transcription profiles due to in vitro supply of methionine with or without choline in unstimulated blood polymorphonuclear leukocytes from lactating Holstein cows. J. Dairy Sci..

[73] Halliwell B., Gutteridge J.M. (2015). Free Radicals in Biology and Medicine.

[74] Nathan C., Ding A. (2010). Nonresolving inflammation. Cell.

[75] Liu J., Han X., Zhang T., Tian K., Li Z., Luo F. (2023). Reactive oxygen species (ROS) scavenging biomaterials for anti-inflammatory diseases: From mechanism to therapy. J. Hematol. Oncol..

[76] Bai H., Shabur T.M.A., Kunii H., Itoh T., Kawahara M., Takahashi M. (2019). Evaluation of the immune status of peripheral blood monocytes from dairy cows during the periparturition period. J. Reprod. Dev..

